# Distributive stress: individually variable responses to hypoxia expand trophic niches in fish

**DOI:** 10.1002/ecy.3356

**Published:** 2021-05-04

**Authors:** Tyler R. Steube, Matthew E. Altenritter, Benjamin D. Walther

**Affiliations:** ^1^ Department of Life Sciences Texas A&M University—Corpus Christi 6300 Ocean Drive Corpus Christi Texas 78412 USA; ^2^ Department of Environmental Science & Ecology The College at Brockport, State University of New York 350 New Campus Drive Brockport New York 14420 USA

**Keywords:** environmental stress, food webs, marine, otolith chemistry, stable isotopes

## Abstract

Environmental stress can reshape trophic interactions by excluding predators or rendering prey vulnerable, depending on the relative sensitivity of species to the stressor. Classical models of food web responses to stress predict either complete predator exclusion from stressed areas or complete prey vulnerability if predators are stress tolerant. However, if the consumer response to the stress is individually variable, the result may be a distributive stress model (DSM) whereby predators distribute consumption pressure across a range of prey guilds and their trophic niche is expanded. We test these models in one of the largest hypoxic “Dead Zones” in the world, the northern Gulf of Mexico, by combining geochemical tracers of hypoxia exposure and isotope ratios to assess individual‐level trophic responses. Hypoxia‐exposed fish occupied niche widths that were 14.8% and 400% larger than their normoxic counterparts in two different years, consistent with variable displacement from benthic to pelagic food webs. The degree of isotopic displacement depended on the magnitude of hypoxia exposure. These results are consistent with the DSM and highlight the need to account for individually variable sublethal effects when predicting community responses to environmental stress.

## Introduction

**Fig. 1:**
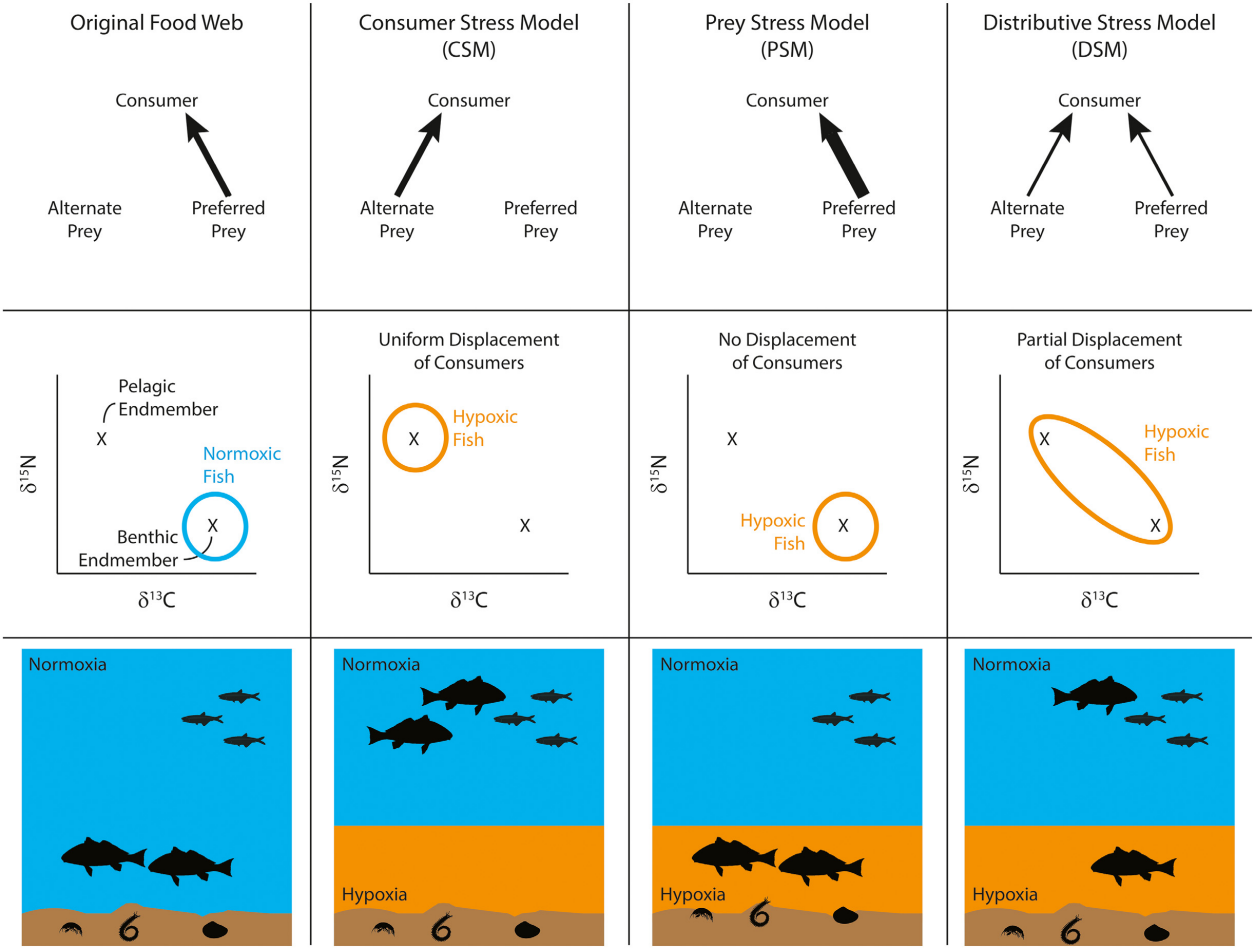
Schematic diagram illustrating predicted trophic responses of animals to physiological stress from hypoxia. Rows show predicted food web topology (arrow thickness representing relative foraging rates), predicted isotope ellipse patterns, and predator distribution in the presence or absence of a benthic hypoxic layer. Columns indicate predictions for the original food web, the consumer stress model (CSM), the prey stress model (PSM), and the distributive stress model (DSM). Isotope schematic plots are modified from Mohan and Walther (2016). Silhouettes were obtained from phylopic.org and used under a Public Domain and Creative Commons licenses (see Appendix S2).

Environmental stress can restructure food webs by modifying predator or prey behaviors, excluding specific guilds from stressed habitats, and providing refuges for species that tolerate higher levels of stress. Physiological tolerances, differential behavioral responses, and heterogeneous exposure to variable environmental stressors are strong mediators of ecosystem processes and evolutionary outcomes in the face of continued perturbations (Killen et al. 2013, Melbinger and Vergassola 2015). Classical paradigms that describe altered predator–prey interactions invoke environmental stress models, which predict divergent outcomes depending on whether the predator or prey is relatively more tolerant of stressful conditions (Menge and Sutherland 1987, Bruno et al. 2003). Two primary models have been delineated: the consumer stress model (CSM), where sensitive predators are excluded from stressful environments that tolerant prey use as refuges, or the prey stress model (PSM), where stress‐tolerant predators continue foraging in habitats where stressed prey are readily consumed (Fig. 1). However, both models assume uniform responses of predators to the environmental stress.

Although ecologists have long recognized that intraspecific variations in traits are critical drivers of niche widths, ecological stability, and evolutionary processes (Van Valen 1965, Roughgarden 1972), ecological theory historically ignored intraspecific trait variation until recently (Araújo et al. 2011, Bolnick et al. 2011, Des Roches et al. 2018). Individual variability may manifest in a variety of ecological processes that are based on genetic diversity or the plastic expression of phenotypic traits driven by heterogeneous experiences. Organisms may differ in their metabolic rate and energy allocation (Careau et al. 2008), sensory perception and responses to stimuli (Leavell and Bernal 2019), assessments of risk (Peacor et al. 2020), and diet specialization (Melián et al. 2014). Optimal foraging theory predicts utilization of the relatively most valuable portion of the resource pool; however, divergence in valuation criteria driven by alternate phenotypic traits can promote unique resource specialization patterns at the individual level. As a result, individual specialization may lead to generalist foraging portfolios that broaden the realized niche at the population level and affect the magnitude of niche overlap and stability of the community (Svanbäck and Bolnick 2007, Bolnick et al. 2011).

Individual variation in biotic interactions, such as predation and competition, can be major drivers of niche breadth at the population level. For instance, assessments of predation risk within a landscape of fear can be highly divergent and repeatable among behavioral syndromes leading to individual dietary specializations on a subset of the potential available resource pool depending on risk tolerance (Gaynor et al. 2019, Steinhoff et al. 2020). Competitive strengths may also vary individually, leading to diverse outcomes in both intraspecific and interspecific competitive interactions. Biotic interactions may thus be instrumental in driving individual patterns of resource use that modify the strength and direction of energy flow among ecological communities.

Most of the recent work quantifying individual variability in food web interactions and diet specialization has focused on biotic drivers of individual trait variation; however, the role of environmental stress in driving trait variation, such as diet specialization, is less well known (Dall et al. 2012). This is often because of practical limitations on the ability of researchers to observe or reconstruct environmental histories for individuals over specific life stages (Stamps and Groothuis 2010). Importantly, the exposure to abiotic conditions is rarely quantified at the individual level and subsequently paired with trait measurements in order to assess impacts of abiotic stress on individual specialization (Lunghi et al. 2020). Individual variation in the exposure, sensitivity, or behavioral response to an environmental stress may lead to a distributive stress model (DSM). The DSM predicts that only some predators are displaced to alternative prey whereas others remain foraging on their preferred prey. This incomplete displacement results in a partial reduction in consumption rates on the preferred prey guild but a proportional increase in consumption rates on an alternative prey guild. As a result, the environmental stress leads to distributed consumer pressure across multiple prey guilds, with this distribution driven by individually variable responses to the stress (Fig. 1). This model predicts altered food web topology for both number and strengths of connections, leading to an expansion of the trophic niche for predators at the population level. There are numerous potential mechanisms by which individually variable traits may alter ecological function, ranging from nonlinear interactions to selection and drift (Bolnick et al. 2011). The DSM specifically predicts altered food web networks with an “increased degree” of connectivity among nodes, even while particular links themselves may weaken in magnitude (Dunne et al. 2002).

A notable stress in marine and aquatic habitats worldwide is the severe decline in dissolved oxygen, or deoxygenation, that can lead to major restructuring of ecosystems (Breitburg et al. 2018). Hypoxia (typically defined as dissolved oxygen <2.0 mg/L) is most acute in coastal or enclosed water bodies that receive significant amounts of anthropogenic nutrient loading that fuel blooms and decomposition of primary producers, which leads to the depletion of bottom water oxygen concentrations (Fennel and Testa 2019). The magnitude of hypoxia impacts depends on the severity, duration, and frequency of oxygen depletion as well as the relative sensitivity of species to oxygen availability (Bickler and Buck 2007, Mandic et al. 2009). Hypoxic zones can be spatially patchy, meaning exposure likelihood may be unpredictable for organisms. Although some organisms with low tolerance to hypoxia may experience outright mortality, others with higher tolerance may survive exposure if the oxygen depletion is moderate in magnitude or brief in duration. Despite surviving hypoxia exposure, significant sublethal effects include altered metabolic scopes, growth rates, condition, and even impaired gamete production (Brandt et al. 2009, Thomas and Rahman 2012, Altenritter and Walther 2019). Additionally, mobile organisms may escape hypoxic waters laterally or vertically. This movement may allow survival but can displace organisms from their preferred prey, concentrate foragers at the edges of hypoxic zones, and lead to altered trophic or competitive interactions in the newly occupied habitats (Breitburg et al. 1997).

The suite of hypoxia impacts ranging from mortality to disrupted physiology to altered trophic interactions can induce major shifts in ecosystem functioning and stability (Wu 2002, Diaz and Rosenberg 2008). The relative sensitivity of predators and prey to hypoxia will be a strong mechanism mediating consumer pressure as predicted by environmental stress models (Fig. 1). For instance, the CSM may apply in hypoxic bottom waters that exclude sensitive predators and allow benthic prey to escape consumption as predators are displaced to pelagic prey (Pihl et al. 1991, Craig and Bosman 2013). This may result in significant reductions in habitat availability for predators and an increase in local densities because of habitat compression (Stramma et al. 2012, Casini et al. 2016). Furthermore, displacement and concentrations of foragers can alter their susceptibility to directed harvest or unintended bycatch by fisheries (Breitburg 2002, Thambithurai et al. 2020). Conversely, the PSM model may apply when benthic infaunal prey evacuate anoxic sediments and are accessible at sediment surfaces where hypoxia‐tolerant fishes may enhance their foraging rates (Long and Seitz 2008, Mohan and Walther 2016). In this case, predators may retain access to their preferred habitats and prey; however, the sublethal effects of hypoxia on physiological processes such as growth and reproduction may have long‐term impacts on population sustainability (Rose et al. 2009). Individually variable responses to hypoxia could lead to an overall expansion of the population‐level trophic niche if some individuals continue to forage on benthic items while others are displaced into pelagic food webs (Fig. 1). If consumer pressure is split among these different prey guilds, then the population‐level trophic niche will expand, and the DSM applies. Importantly, individually heterogeneous exposure and behavioral responses to an environmental stressor may be critical for determining long‐term evolutionary adaptation and alterations to trait variation distributions in the face of hypoxia (Killen et al. 2012, Norin et al. 2016). Individually variable responses to exposure can, therefore, result in complex ecosystem dynamics in these stressed systems (Bergman et al. 2019).

We examined changes in trophic interactions resulting from exposure to deoxygenated bottom waters in the northern Gulf of Mexico (nGoMex), the second largest anthropogenically derived hypoxic region in the world (Rabalais and Turner 2019). Lifetime hypoxia exposure can be reconstructed for individuals using redox‐sensitive elements in fish ear stones (otoliths; Limburg et al. 2015, Limburg and Casini 2019), whereas trophic niche breadth can be assessed with muscle tissue stable isotopes (Hammerschlag‐Peyer et al. 2011). Direct links between hypoxia exposure and trophic responses were made by combining these two sets of proxies for individual fish. A hypoxia‐induced shift from benthic to pelagic prey is expected to result in altered isotopic niches given observed differences of 2–5‰ in δ^13^C between benthic and pelagic food webs across numerous systems worldwide (Peterson 1999, Davenport and Bax 2002) and specifically on the order of 2–3‰ between benthic and pelagic baselines in the nGoMex (Radabaugh and Peebles 2014). For this study we used Atlantic croaker (*Micropogonias undulatus*), given its preference for feeding on benthic prey such as polychaetes and bivalves but also the ability to switch to pelagic prey such as anchovies (Nye et al. 2011). As a result, δ^15^N values in hypoxia‐displaced Atlantic croaker may increase with the switch to pelagic prey, although continued feeding on higher trophic levels within benthic communities (small demersal fishes and crustaceans) could complicate the response of δ^15^N values to hypoxia. Using a combined otolith and tissue isotope proxy approach for multiple years, we evaluated whether hypoxia exposure across 2 yr consistently resulted in (1) a uniform response indicating all hypoxia‐exposed individuals were displaced from their normoxic isotopic niche (consistent with a CSM), (2) no trophic response indicating resilience and continued benthic foraging (consistent with a PSM), or (3) a variable response with only some individuals shifted in their isotope values, resulting in an expansion of the cumulative isotopic niche for hypoxia‐exposed fish (consistent with a DSM). For the DSM model, we specifically expect niche expansion in δ^13^C with more individuals exhibiting lower isotope values.

## Methods

### Otolith chemistry

Atlantic croaker were collected in the nGoMex during October and November in 2014 and 2015 aboard the National Oceanographic and Atmospheric Administration’s R/V *Oregon II* using standardized benthic trawls conducted for the Southeast Monitoring and Assessment Program’s autumn groundfish surveys (Altenritter and Walther 2019). Trawls were conducted at stations within the typical hypoxic zone that forms annually during summer months off the coast of Louisiana (Appendix S1: Fig. S1). The hypoxic zone in the nGoMex develops over summer typically reaching a maximum areal extent in August–September before the onset of winter storms begins to reaerate the water column (Rabalais et al. 2002). Prior experiments validating isotope turnover rates for Age‐0 Atlantic croaker established that 95% equilibration of muscle tissue isotopes occurred in 115 ± 12 d for δ^15^N and 129 ± 47 d for δ^13^C (Mohan et al. 2016). In addition, Atlantic croaker otoliths from the same experiment accreted an average of 1,000 μm along the standard ablated axis over a 3‐month period, meaning the exterior portion of the otolith can be matched to the same time period integrated by tissue isotopes (Mohan and Walther 2016). Thus, collections that occurred in October and November contain fish with tissue and otolith chemical compositions that reflect feeding patterns and hypoxia exposure patterns of the prior 3–4 months overlapping the maximal hypoxic period in the region for Age‐0 Atlantic croaker.

All individuals collected were stored frozen at −20°C until processing. Fish were dissected and sagittal otoliths and dorsal white muscle tissue samples of 1–2 g in mass (excluding skin and scales) were removed from all individuals. The left sagittal otolith from each individual was rinsed with deionized water, allowed to air dry under a class‐100 laminar flow hood, then embedded in epoxy, thick sectioned (1–2 mm) along the transverse plane, and polished sequentially with 30‐μm and 3‐μm lapping films until the core was visible. Sectioned otoliths were independently aged by two readers to identify the Age‐0 fish used for this study (Altenritter et al. 2018). The ratios of otolith Mn:Ca (hypoxia proxy) and Ba:Ca (salinity proxy differentiating offshore and inshore habitats) were measured using laser ablation inductively coupled mass spectrometry in the Jackson School of Geosciences at the University of Texas at Austin. Otoliths were ablated using an Agilent 7500ce ICP quadrupole mass spectrometer coupled to a New Wave UP 193‐FX laser. Full analytical details are provided by Altenritter and Walther (2019). Following a preablation (spot diameter = 50 μm; scan rate = 50 μm/s) to remove surface contaminants, element concentrations in otoliths were measured along a transect spanning the otolith core to the edge along the longest growth axis (spot diameter = 25 μm; scan rate = 5 μm/s). Two standard reference materials (NIST‐612, USGS MACS‐3) were run in triplicate after every 5–6 otoliths to correct for drift, assess analytical precision, and convert intensity readings to molar concentrations relative to calcium. Mean relative standard deviations over all analytical days were 5.2% for Mn:Ca and 3.8% for Ba:Ca.

Otolith transects were truncated to the exterior 1,000‐μm measured to obtain an estimated 3 months of life prior to capture (Mohan and Walther 2016). This time frame matches the amount of time integrated by muscle isotope ratios given experimentally validated turnover rates for this species (Mohan et al. 2016). An indexing and clustering procedure was used to identify Age‐0 Atlantic croaker that experienced hypoxia during recent months in offshore nGoMex habitats based on measured otolith element ratios. Briefly, exceedance thresholds of otolith Mn:Ca and Ba:Ca were established based on (1) previous field‐derived relationships between dissolved oxygen and waterborne Mn, and between salinity and waterborne Ba; and (2) laboratory experiments with Atlantic croaker quantifying otolith Mn:Ca and Ba:Ca incorporation dynamics (Mohan et al. 2014, Mohan and Walther 2016, Altenritter et al. 2018). Measured otolith values above established exceedance thresholds for Mn and Ba indicated exposure to hypoxia and inshore estuarine habitats respectively. Otolith values across the transect were ascribed values of 1, 2, or 3 where measured values exceeded thresholds by 1, 2, or 3 times. A simultaneity index (joint exceedance of Mn:Ca and Ba:Ca thresholds) was used to identify and exclude individuals that may have experienced hypoxia in inshore estuarine environments given our focus on hypoxia exposure in offshore environments. Hypoxia, estuarine, and simultaneity index values for each individual were used in a Ward’s hierarchical cluster analysis to separate groups based on past exposure history. This left 84 fish collected in 2014 and 73 fish collected in 2015 that were classified as either offshore hypoxic or offshore normoxic. As estuarine resident fish were not the target of the current study, only offshore normoxia‐ and hypoxia‐exposed fish were used for subsequent isotope niche comparisons.

### Stable isotopes

White muscle tissue samples from the selected Age‐0 hypoxic and normoxic fish were dried for 48–72 h at 60°C, ground with a mortar and pestle, and approximately 1 mg of powdered tissue was packed in tin capsules prior to elemental analysis and stable isotope analysis at the University of Texas Marine Science Institute. Elemental (C:N) and isotope (δ^15^N and δ^13^C) ratios were quantified using a Carlo Erba NC2500 elemental analyzer and a Thermo Delta V Plus isotope ratio mass spectrometer. Daily standard calibrations using certified reference materials USGS 40 (l‐glutamic acid), USGS 41a (l‐glutamic acid enriched in ^13^C and ^15^N), and an internal laboratory standard of peach leaves were performed before, during, and after stable isotope analysis runs. Isotope ratio results were reported in standard delta‐notation (‰) relative to AIR for δ^15^N and Vienna Pee Dee Belemnite for δ^13^C. Instrumental precision was 0.05‰ for δ^13^C and 0.07‰ for δ^15^N based on repeated measurements of certified reference USGS‐40. Duplicates for every 20th fish muscle sample revealed average precisions of 0.05‰ for δ^13^C and 0.03‰ for δ^15^N values.

Linear regressions were calculated for δ^13^C and δ^15^N values against fish standard length (mm) and C:N ratios to verify if any size or lipid content–related trends in isotope values may bias comparisons between hypoxic and normoxic groups (Post et al. 2007). There was no significant relationship between δ^15^N and C:N ratios (*P* = 0.21) and a marginally significant relationship with standard length (*P* = 0.05 with a low adjusted *R* ^2^ of 0.02). No adjustment was performed for δ^15^N given the marginally significant and weak relationship between isotope values and standard length. There was no significant relationship between δ^13^C and C:N ratios (*P* = 0.44); however, a significant relationship was found between δ^13^C and standard length (*P* < 0.01; adjusted *R* ^2^ = 0.13). Adjusted δ^13^C values were calculated by normalizing with respect to the global δ^13^C versus length relationship to remove the potential bias effect of length on isotope values, analogous to procedures used to detrend whole otolith chemical analyses to remove effects of mass (Campana et al. 2000, Kerr and Campana 2014). Length‐adjusted δ^13^C values were used for all subsequent analyses.

Based on otolith chemistry results, fish were classified as exposed to either offshore hypoxia or normoxia prior to capture. Tissue isotope ratios were then compared between these two exposure groups for each year of capture. Univariate comparisons of isotope ratios between exposure groups were made with nonparametric Mann‐Whitney rank sum tests. Bivariate analyses of both isotope ratios together were made by calculating Bayesian standard ellipses encompassing approximately 40% of the data using the software package SIBER (Jackson et al. 2011). In addition, maximum‐likelihood estimates of the standard ellipse area corrected for sample size (SEAc) as well as 50%, 75%, and 95% credible intervals of the standard ellipse areas were calculated for each exposure group and collection year. In order to investigate within‐group variability for the hypoxia‐exposed fish alone, year‐specific linear regressions were calculated separately for individual pairwise values of the tissue δ^13^C and δ^15^N values against the hypoxia‐exposure index value derived from otolith chemistry, where a higher hypoxia index value indicates higher hypoxia‐exposure magnitude and duration.

## Results

**Fig. 2:**
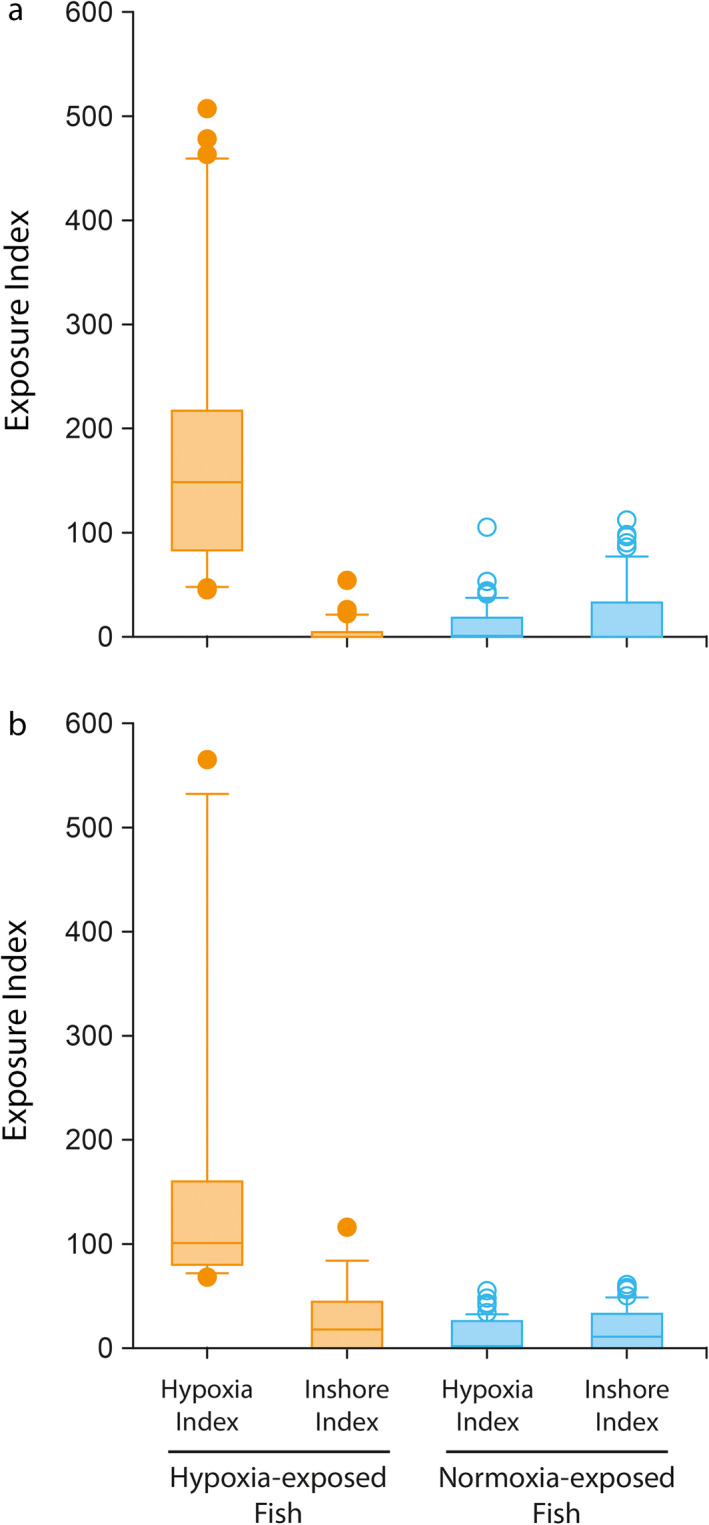
Exposure indices derived from otolith chemical tracers indicating exposure to hypoxia or inshore estuarine habitats prior to capture in (a) 2014 and (b) 2015 in the northern Gulf of Mexico. Fish identified as hypoxia exposed (closed orange symbols) had high hypoxia index values and low inshore index values, reflecting their interactions with the offshore “Dead Zone” region. Fish identified as normoxia exposed (open blue symbols) had low values of both hypoxia and inshore indices, reflecting their occupancy of fully oxygenated nonestuarine offshore waters.

Standard lengths of collected Age‐0 fish were 108 ± 1 mm (1 standard error) in 2014 and 115 ± 1 mm in 2015. Fish masses were 33.6 ± 0.9 g in 2014 and 36.2 ± 0.9 g in 2015. Otolith chemistry identified fish that were classified as hypoxia‐exposed or normoxia‐exposed prior to capture based on a combination of high Mn:Ca ratios and low Ba:Ca ratios (Appendix S1: Fig. S2). Mean hypoxia indices for normoxia‐exposed fish were 12 ± 3 (1 SE) in 2014 and 12 ± 2 in 2015, and indices for hypoxia‐exposed were 185 ± 25 in 2014 and 162 ± 37 in 2015 (Fig. 2). Hypoxic fish made up 34% (*n* = 30) of the total fish analyzed, including estuarine‐exposed fish, in 2014 and 18% (*n* = 17) of the total in 2015, whereas normoxic fish made up 61% (*n* = 54) of the total in 2014 and 59% (*n* = 56) of the total in 2015 (Appendix S1: Fig. S3). Excluding estuarine‐exposed fish, hypoxic fish made up 36% of the remaining individuals in 2014 and 23% of the remaining individuals in 2015.

**Fig. 3:**
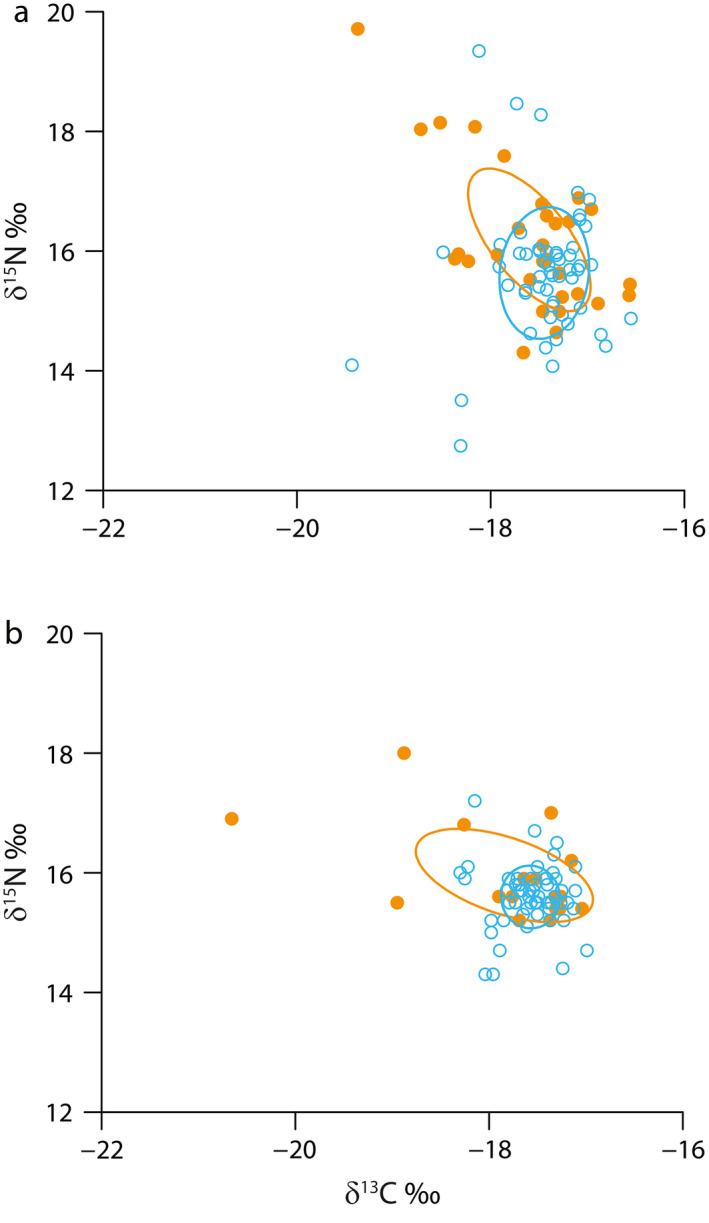
Isotope values for Age‐0 Atlantic croaker identified as hypoxia exposed (closed orange symbols) or normoxia exposed (open blue symbols) prior to capture in (a) 2014 and (b) 2015 in the northern Gulf of Mexico. Bayesian‐inferred standard ellipses are shown for each exposure type.

**Fig. 4:**
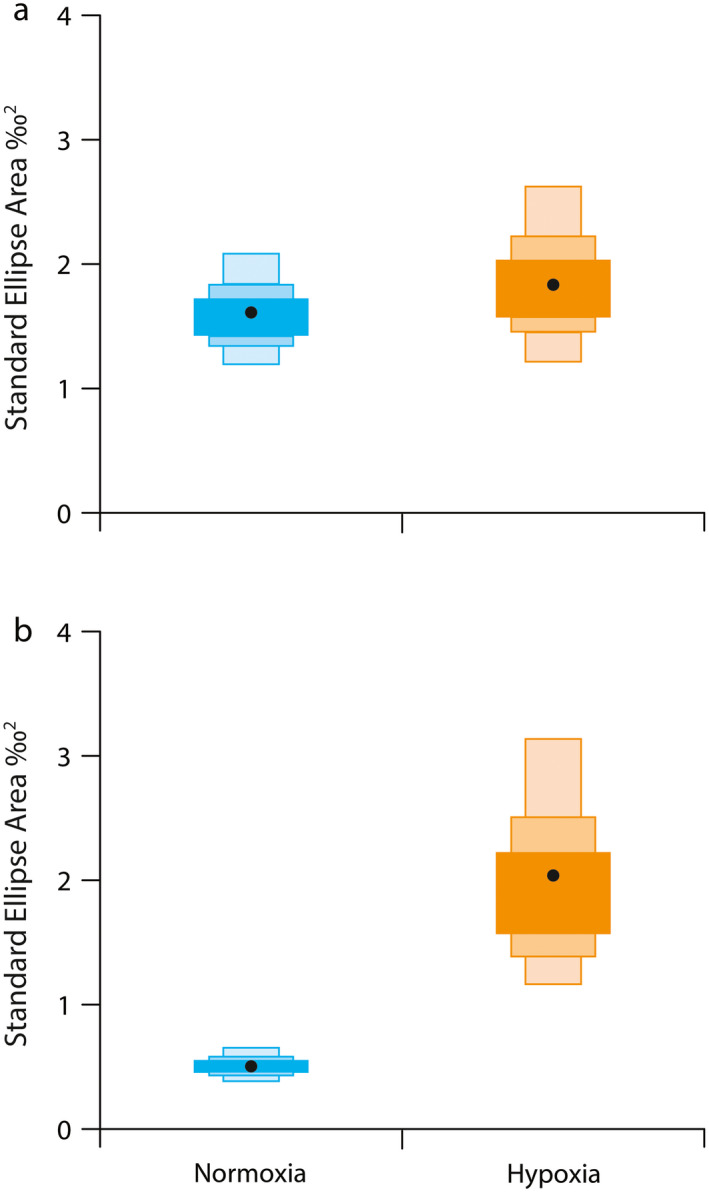
Bayesian‐estimated standard ellipse areas for Age‐0 Atlantic croaker exposed to hypoxia or normoxia prior to capture in (a) 2014 and (b) 2015 in the northern Gulf of Mexico. The black circle is the maximum‐likelihood estimate of the standard ellipse area after correction for small sample sizes (SEAc). Shaded boxes represent 50%, 75%, and 95% credible intervals of ellipses from dark to light shades, respectively.

There was substantial overlap in isotope values between normoxic and hypoxic fish in both years (Fig. 3). Univariate differences in either δ^13^C or δ^15^N isotope values between hypoxic and normoxic fish were not statistically significant in either 2014 (*P* = 0.264 for δ^13^C, *P* = 0.344 for δ^15^N) or 2015 (*P* = 0.906 for δ^13^C, *P* = 0.059 for δ^15^N). This was reflected in ellipse overlap and similar mean values of δ^13^C and δ^15^N in hypoxic and normoxic fish in both years (Appendix S1: Table S1). However, standard ellipses for hypoxic fish were elongated, with relatively more individuals having higher δ^13^C and δ^15^N values compared to normoxic fish. These individuals had δ^13^C values that were 2–3‰ lower than most normoxic fish values, as expected. As a result, standard ellipse area estimates were larger for hypoxic fish in both years (Fig. 4). Values of SEAc were 1.62‰^2^ for normoxic fish and 1.86‰^2^ for hypoxic fish in 2014, and values were 0.51‰^2^ for normoxic fish and 2.04‰^2^ for hypoxic fish in 2015. Isotopic niche area estimates for hypoxic fish were, therefore, 14.8% and 400% larger than normoxic fish niche areas in 2014 and 2015, respectively.

**Fig. 5:**
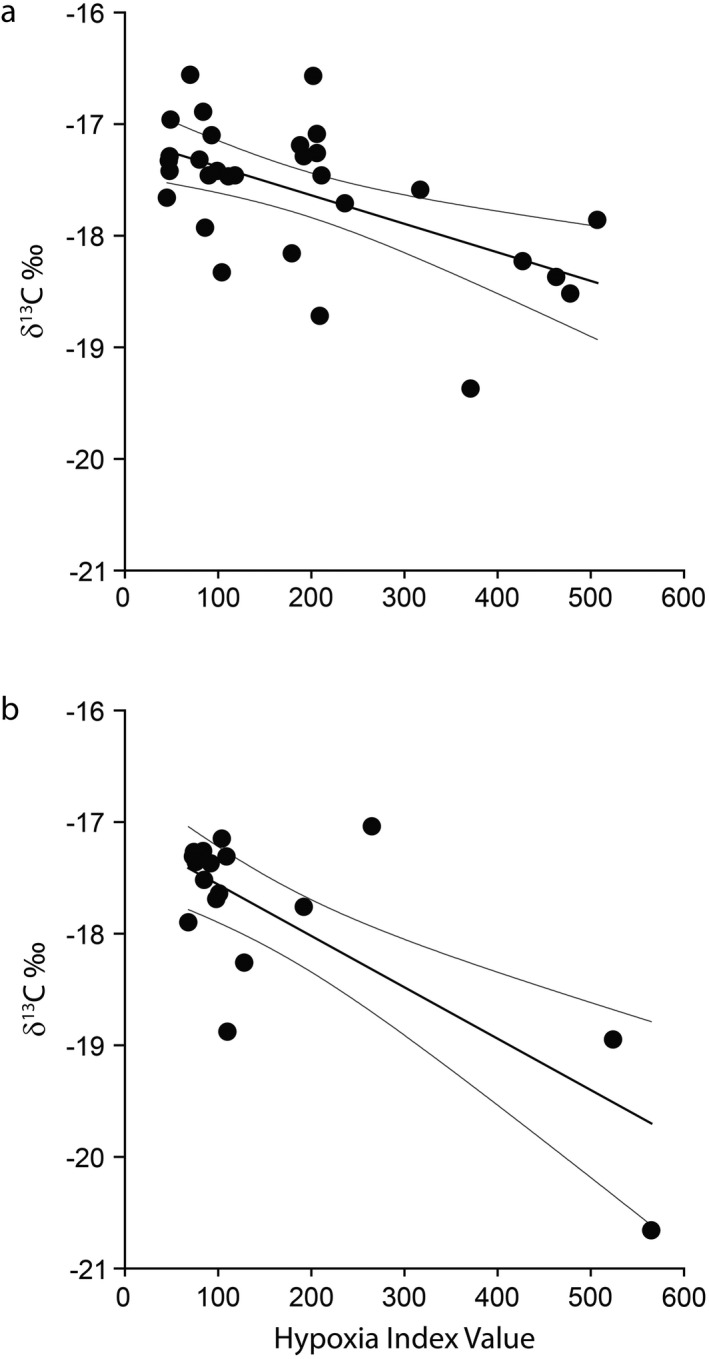
Linear regressions and 95% confidence intervals of muscle tissue δ^13^C values and otolith edge hypoxia index values derived from otolith Mn:Ca ratios from Age‐0 Atlantic croaker captured in (a) 2014 and (b) 2015 in the northern Gulf of Mexico. Only fish identified as hypoxia‐exposed individuals based on otolith chemistry are shown.

Linear regressions between δ^15^N values and otolith hypoxia index value were statistically significant in 2014 (*P* = 0.01, *R* ^2^ = 0.21) but not in 2015 (*P* = 0.71, *R* ^2^ = 0.01). The slope for the regression in 2014 was positive (slope of 0.004‰ ± 0.001 [1 SE], intercept of 15.449‰ ± 0.331). Linear regressions between tissue δ^13^C values and otolith hypoxia index values for hypoxia‐exposed individuals were statistically significant in both 2014 (*P* = 0.001, *R* ^2^ = 0.32) and 2015 (*P* < 0.001, *R* ^2^ = 0.59). Both regressions had a negative slope, indicating lower δ^13^C values as hypoxia exposure increased (Fig. 5). Regression equations for each year differed (slope of −0.003‰ ± 0.001, intercept of −17.125‰ ± 0.163 for 2014; slope of −0.005‰ ± 0.001, intercept of −17.099‰ ± 0.218 for 2015), with a steeper slope and lower intercept in 2015. However, parameter estimates were within 1 SE of each other, suggesting broadly similar relationships despite interannual variability in hypoxia extent and sample size differences.

## Discussion

Individual responses to biotic interactions and abiotic conditions can significantly impact the function and resilience of populations in spatially and temporally heterogeneous ecosystems. The ecological drivers of individual trait variation can be manifold and simultaneous, thereby complicating predictions of population and community dynamics in response to shifting conditions. Most of the recent empirical and theoretical evaluations of the impacts of individual variation have focused on biotic interactions such as predation and competition. Assessing the magnitude of within‐population niche variation requires variance partitioning of resource use among and between individuals. One metric for individual specialization is the ratio of variance in individual resource use (WIC) to total niche width of the population (TNW; Bolnick et al. 2002). In a recent review, Araújo et al. (2011) found 189 species in which some degree of individual specialization was quantified and individual niches were on average only 66% of the corresponding population niches. These included instances across taxonomic groups from plants to mammals, with fishes constituting the largest represented group. Whereas these patterns may be subject to sampling bias and underpublication of the absence of individual specialization, the possibility that certain taxa are more likely to exhibit individual specialization could have important implications for the stability and functioning of complex communities.

The role of intraspecific trait variation on ecological performance has been debated for decades (Des Roches et al. 2018). Although traditional optimal foraging theory assumed that population niche widths were reflected by individual niche widths given shared dietary optima (Pyke et al. 1977, Jesmer et al. 2020), the niche variation hypothesis predicts individual variation in dietary specialization collectively contributes to a wider population‐level niche width (Van Valen 1965, Roughgarden 1972). Although the importance of individual dietary specialization has been increasingly identified and quantified, the ecological drivers of specialization can be difficult to identify. The interplay between environmental stress and ecosystem responses can result in alterations in food web connectance, stability, and the prevalence of omnivory (Gibert 2019). Stressors may modulate ecological interactions that facilitate the expression of individually variable responses and ultimately impact community structure and function across multiple biological scales (Start and Gilbert 2019). These stressors may include abiotic conditions, including habitat configuration and temperature. For example, trophic niche expansion in *Gambusia hubbsi* residing in fragmented, low‐predation‐risk habitats was associated with increased intraspecific competition and potential behavioral shifts that allowed access to previously unavailable food resources (Araújo et al. 2014). Individual variation in vital rates (e.g., growth and metabolism) are also anticipated to influence trophic responses to stressors such as warming water temperatures, which could act differentially on individuals given size‐dependent links to food intake and predator–prey interactions (Gårdmark and Huss 2020). The observation that stress has the potential to modify trophic dynamics based on individual variation in response underpins the need to evaluate exposure at that level. However, there is a relative paucity of information about individual stress histories from wild‐captured specimens. Otolith chemistry as used here provided knowledge of exposure history to hypoxia at the individual level and generally holds promise as a link between additional stressors and outcomes for trophic dynamics.

The impacts of declining oxygen in global waters can manifest at multiple ecological levels, leading to strong linkages along an environment–physiology–demographic continuum (Bergman et al. 2019). Here, we quantified individually variable rates of exposure to offshore hypoxia in a common mobile fish using intrinsic geochemical tracers and couple those with isotope proxies of food web interactions. We found that sublethal exposure varies among individuals and across years. The proportions of fish that are exposed to hypoxia and survive can range from one‐fifth to one‐third of analyzed fish, consistent with prior estimates for this species (Mohan and Walther 2016, Altenritter et al. 2018, Altenritter and Walther 2019). Sublethal exposure can be a critically important factor that determines future population productivity, especially given hypoxic effects on growth, condition, metabolism, and reproductive output (Rose et al. 2018). The magnitude of sublethal exposure rates will differ among species within a single system as well, given species‐specific oxygen tolerances, avoidance behaviors, and flexibility in habitat and dietary specialization. Individuals within a population may also exhibit divergent responses depending on their exposure magnitude, prior acclimation, and ontogenetic stage. Thus, hypoxia as a stressor will not act uniformly on species and individuals within a system but may instead elicit a range of responses that lead to differential performance and functional interactions.

In addition, we found that the magnitude of the niche expansion varied by year, perhaps driven by environmental variability in the size and spatial patchiness of the bottom‐water hypoxic zone in the northern Gulf of Mexico. For instance, standardized summer assessments in the Gulf of Mexico reported that the spatial coverage of bottom‐water hypoxia increased 128% from 2014 (13,080 km^2^) to 2015 (16,760 km^2^; Rabalais and Turner 2020). Concordantly, we observed that the isotopic niche of hypoxia‐exposed fish was 400% larger than the niche of normoxic fish in 2015, but only 14.8% larger than the niche of normoxic fish in 2014. A previous study by Mohan and Walther (2016) on Atlantic croaker captured in 2011 and 2012 found that hypoxic and normoxic niche sizes were nearly identical (areas of 2‰^2^ and 3‰^2^ for hypoxic and normoxic fish, respectively), indicating limited displacement and continued benthic foraging. However, those analyses included fish from 2012, which had one of the smallest recorded northern Gulf of Mexico hypoxic spatial coverage recorded in recent years (7,480 km^2^). Interannual variability in environmental drivers that alter the size and extent of hypoxic bottom waters are likely important drivers in the temporal fluctuations of species responses within a system.

The isotopic niches of hypoxic‐exposed fish were expanded and shifted primarily by altered δ^13^C that were 2–3‰ lower than their normoxic counterparts. This magnitude of this shift in the carbon isotope ratio is consistent with a displacement away from benthic‐type isotope values and towards pelagic‐type values (Wang et al. 2018). Ideally, tissue isotope values are paired with complementary assessments of diet choice, such as gut content analysis or visual observation of foraging. These data were not available in the current study, because fish were retrieved with trawls, and most specimens apparently had evacuated stomach contents during net retrieval (Mohan and Walther 2016). Future samples of potential prey items and isotopic baselines could help confirm that the observed isotopic shifts fully reflect the benthic to pelagic displacements. However, several lines of evidence support the interpretation of these isotopic shifts as indicative of benthic displacement. First, bulk organic carbon δ^13^C values in surficial sediments of the nGoMex are typically around −22‰ (Mayer et al. 2007), although dissolved inorganic carbon fluxing from sediments in the region may have δ^13^C values as high as −14‰ to −17‰ (Berelson et al. 2019). Conversely, pelagic surface water particulate organic carbon in the same region has lower δ^13^C values on the order of −24‰ to −26‰ (Wang et al. 2018). Similarly, pelagic phytoplankton in the nGoMex typically have δ^13^C values on the order of −22‰, and benthic algae are approximately −18‰, and similar magnitudes of differences in δ^13^C values are seen consistently in benthic or pelagic feeding fishes (Jay et al. 2006, Radabaugh and Peebles 2014, Tarnecki and Patterson 2015). Furthermore, Atlantic croaker have been observed to be vertically displaced by benthic hypoxia as well to switch their diet from predominantly benthic infauna to small pelagic fishes in other regions, such as mid‐Atlantic estuaries (Eby and Crowder 2002, Eby et al. 2005, Nye et al. 2011). The responses of δ^15^N values to hypoxia exposure were less consistent, as expected given the potential for benthic foraging fishes to also consume higher trophic position organisms such as small demersal fishes and crustaceans. Together, these arguments support δ^13^C values as robust indicators of benthic or pelagic feeding, especially when coupled with the otolith chemistry intrinsic indicator of hypoxia exposure.

The magnitude of the hypoxia exposure drove the level of carbon isotopic shifts, suggesting that individual exposure patterns affect the likelihood of displacement from benthic food webs. Although these relationships were strongly driven by a few fish with substantially different isotope values, we believe this reflects the reduced likelihood of sampling individuals that experienced severe hypoxia exposure and survived to capture. Although hypoxia is not the sole potential driver of isotopic variability in consumers in dynamic offshore systems, the strong associations between the hypoxia‐exposure index and carbon isotope value indicates this stressor has a strong influence on trophic dynamics for this system. Variable exposure among individuals may be driven by patchy and uneven distributions of hypoxic waters in three dimensions (LaBone et al. 2019) as well as individual responses to low oxygen thresholds and relative preference for benthic prey items. A next step in quantifying shifts in individual foraging traits for hypoxic and normoxic exposed fish would be to quantify WIC/TNW ratios. For the current study, single measurements of tissue isotopes provide proxies of TNW indicating the population‐level niche breadth. Assessing the within‐individual variance in foraging (WIC) will require additional gut content measurements or repeated measures of δ^13^C and δ^15^N values in incrementally growing structures, as has been accomplished with bird feathers and mammal whiskers (Araújo et al. 2007, Robertson et al. 2014, Kernaléguen et al. 2016). Fish eye lenses and scales offer considerable promise for incremental structures that could be subsampled for individual dietary isotope histories, and could be added to the suite of tools assessing impacts of hypoxia on individual foraging responses (Wallace et al. 2014, Seeley and Walther 2018).

Prior assessments of food web structural changes in response to hypoxia have interpreted patterns to be consistent with either the prey stress model, whereby prey are more sensitive to hypoxia than predators and thus more susceptible to predation mortality during hypoxia, or the consumer stress model, whereby predators are more sensitive to hypoxia and prey may use hypoxic waters as a refuge from predation (Menge and Sutherland 1987, Long and Seitz 2008, Mohan and Walther 2016). We found significant within‐population variation in responses to hypoxia, which suggests that neither of those models fully applies to the “Dead Zone” in the northern Gulf of Mexico. Instead, diverse responses to hypoxia means a relative shifting of consumption effects across prey groups, but not fully eliminating consumptive pressure from a single group. We call this the DSM (Fig. 1), in which an abiotic stress such as hypoxia elicits individually variable responses that partially alleviate consumptive stress on benthic prey but displace that consumptive stress onto pelagic prey. The portfolio of these complex hypoxia‐driven responses within ecosystems will need to be fully accounted for when evaluating long‐term system‐level resilience to this major stressor.

## Supporting information

Appendix S1

Appendix S2

## Data Availability

Otolith chemistry index and stable isotope data (Walther et al. 2020*a*) are available in Figshare. https://doi.org/10.6084/m9.figshare.12755927.v2 Otolith chemistry raw data (Walther et al. 2020*b*) are available from the Biological and Chemical Oceanography Data Management Office (BCO‐DMO). https://doi.org/10.26008/1912/bco‐dmo.784969.1
