# Supplementary material for: Distributive stress: individually variable responses to hypoxia expand trophic niches in fish
Source: Ecology. 2021 May 4;102(6):e03356. doi: 10.1002/ecy.3356 (PMC8244237; doi:10.1002/ecy.3356)
Supplement: Supplementary file 1 — Appendix S1 [file ECY-102-e03356-s001.pdf]

Supporting Information. Steube, T.R., M.E. Altenritter, and B.D. Walther. 2021. Distributive stress: individually variable responses to hypoxia expand trophic niches in fish. Ecology.

Appendix S1

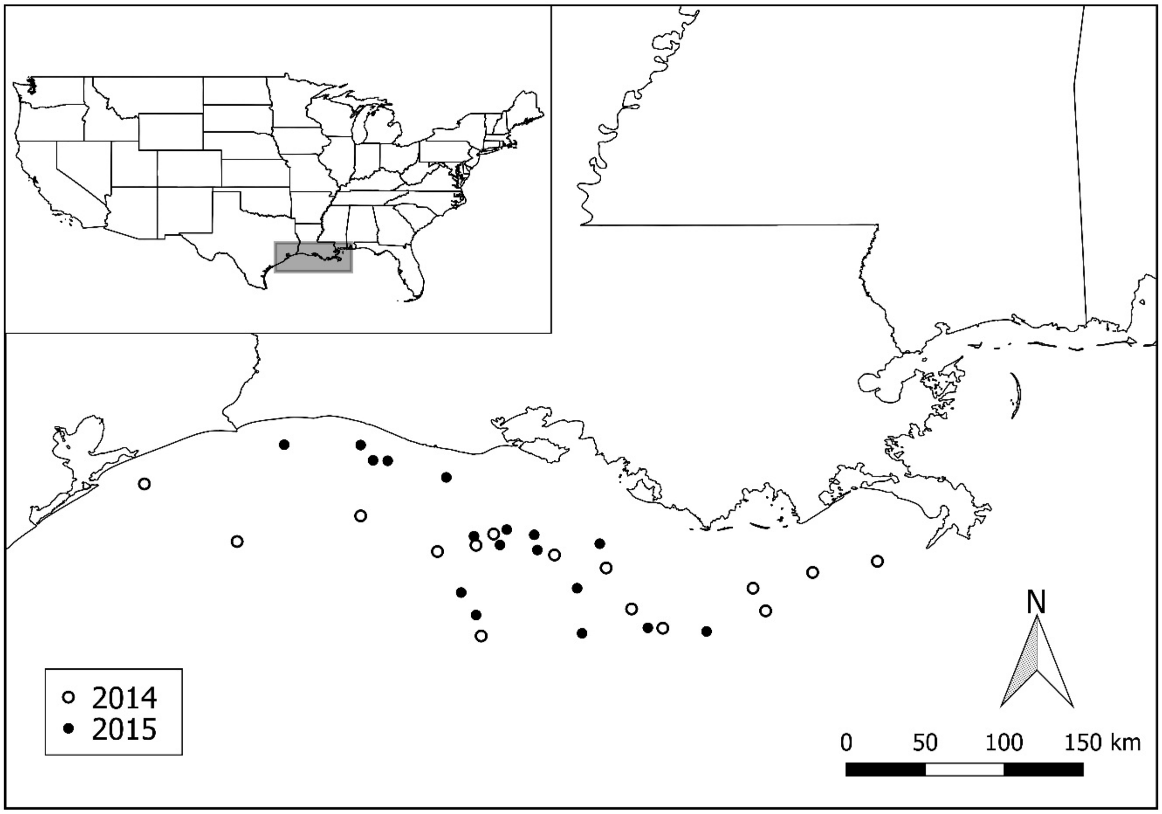

Figure S1. Map of the northern Gulf of Mexico benthic trawl survey locations in 2014 (open circles) and 2015 (closed circles) from which Age-0 Atlantic Croaker were collected.

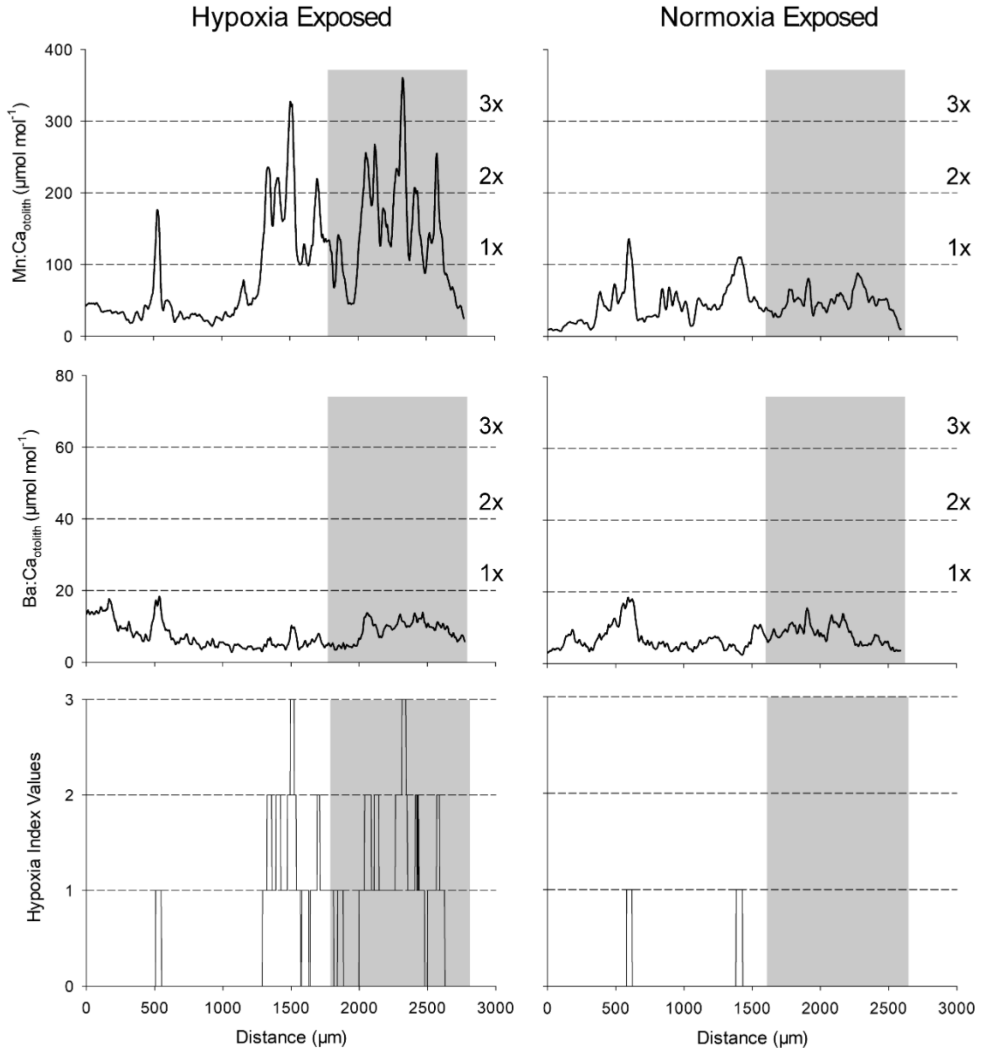

Figure S2. Example laser transects of hypoxia-exposed and normoxia-exposed Age-0 Atlantic Croaker. The transect portions shaded in gray indicate the last 1000µm on the otolith representing three months of life before capture. The dashed lines denoted multiples of the baseline threshold (1x, 2x, and 3x) indicating exposure to hypoxia (Mn:Ca) or inshore environments (Ba:Ca). Each individual was assigned an exposure index value based on the summed threshold exceedance values (1x = 1, 2x = 2, 3x = 3) across the last 1000µm of the otolith. Examples of the hypoxia index values for a hypoxia exposed fish (large hypoxia index value) and a normoxia exposed fish (small hypoxia index value) are displayed.

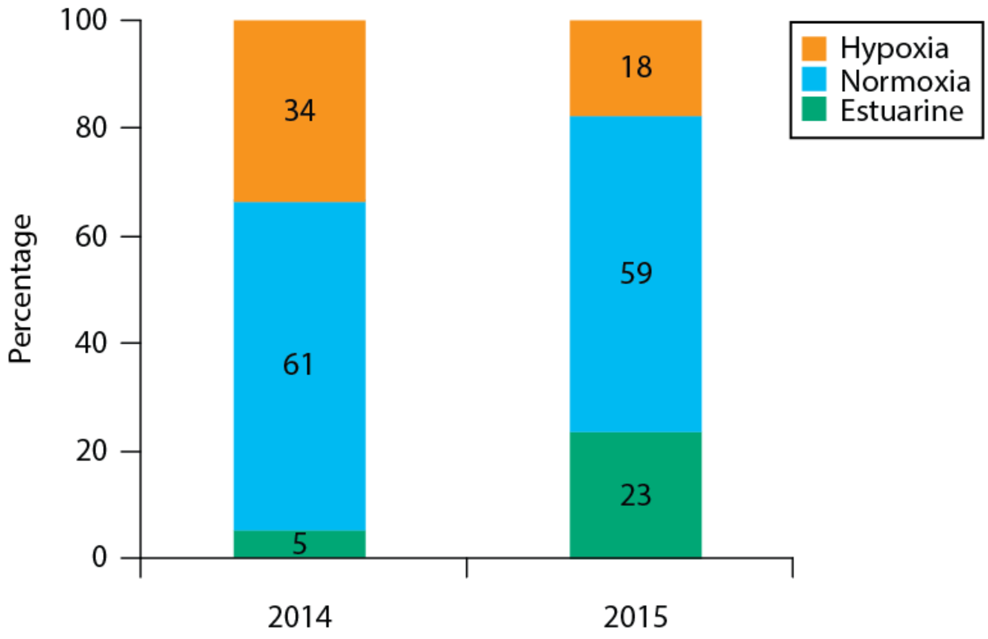

Figure S3. Proportions of fish designated as experiencing hypoxia, normoxia, or estuarine conditions for Age-0 Atlantic Croaker exposed to hypoxia or normoxia prior to capture in 2014 and 2015 in the northern Gulf of Mexico. Exposure histories were determined from the chemical composition (Mn/Ca for hypoxia, Ba/Ca for estuarine) of otolith exterior increments matching the same time period integrated by tissue stable isotopes.

Table S1. Mean (1 standard error) values of $\delta^{13}C$ and $\delta^{15}N$ (‰) in Atlantic croaker muscle tissues from fish identified by otolith chemistry as normoxic or hypoxic exposed and collected in 2014 and 2015.

	$\delta^{13}C$		$\delta^{15}N$	
	2014	2015	2014	2015
Normoxic fish	-17.44 (0.06)	-17.57 (0.04)	15.62 (0.15)	15.58 (0.07)
Hypoxic fish	-17.60 (0.12)	-17.84 (0.22)	16.18 (0.22)	15.95 (0.19)
