# Supplementary material for: Distributive stress: individually variable responses to hypoxia expand trophic niches in fish
Source: Ecology. 2021 May 4;102(6):e03356. doi: 10.1002/ecy.3356 (PMC8244237; doi:10.1002/ecy.3356)
Supplement: Supplementary file 2 — Appendix S2 [file ECY-102-e03356-s002.pdf]

Supporting Information. Steube, T.R., M.E. Altenritter, and B.D. Walther. 2021. Distributive stress: individually variable responses to hypoxia expand trophic niches in fish. Ecology.

Appendix S2

Additional information for Figure 1

Animal silhouettes in Figure 1 were obtained from phylopic.org and used under a Public Domain Dedication License 1.0 (Anchoa by M. Kolmann, Nuculana by K. Collins), Public Domain Mark License 1.0 (Micropogonias, by N. Schooler), Creative Commons Attribution-NonCommercial-ShareAlike 3.0 Unported license (Nereis by B. Duygu Özpolat, https://creativecommons.org/licenses/by-nc-sa/3.0/), and a Creative Commons Attribution-ShareAlike 3.0 Unported license (Leucothoe by Hans Hillewaert, https://creativecommons.org/licenses/by-sa/3.0/). No alterations were made to silhouettes.
